# Anti-fouling activity and toxicity of the Mediterranean alien sponge *Paraleucilla magna* Klautau, Monteiro & Borojevic, 2004 (Porifera, Calcarea)

**DOI:** 10.7717/peerj.12279

**Published:** 2021-10-22

**Authors:** Caterina Longo, Roberta Trani, Carlotta Nonnis Marzano, Maria Mercurio, Tamara Lazic, Pietro Cotugno, Erika Santobianchi, Maria Flavia Gravina

**Affiliations:** 1 Department of Biology, University of Bari, Bari, Italy; 2 Consorzio Nazionale Interuniversitario per le Scienze del Mare (CoNISMa), Rome, Italy; 3 Department of Biology, University of Rome “Tor Vergata”, Rome, Italy

**Keywords:** Antifouling activity, Bioassay, *Paraleucilla magna*, Porifera, Toxicity

## Abstract

Poriferans, as sessile organisms without rigid external covering, use secondary metabolites for protection from predators and fouling organisms. The present study tested the antifouling activity of ethanolic extract of the Mediterranean alien calcareous sponge *Paraleucilla magna* towards juvenile mussels *Mytilus galloprovincialis*. Furthermore, toxicity tests on nauplii of brine shrimp *Artemia salina* and two microalgae strains, *Nannochloropsis* sp. and *Tetraselmis suecica,* were also conducted*.* A total attachment inhibition of *M. galloprovincialis* was achieved at a concentration of 400 µg/mL of sponge extract. The 50% mortality of *A. salina* nauplii was recorded at a concentration of 500 µg/mL of ethanolic extract. The growth inhibitory effect on both marine microalgae strains has been registered at a concentration of 300 µg/mL. Our results suggest promising natural antifouling activity and low toxicity of the ethanolic extract of *P. magna* that could be used as antifouling compound.

## Introduction

Artificial substrata submerged in sea water are rapidly covered by microorganisms and subsequently colonized by sessile invertebrates: all such covering is commonly defined as “biofouling” (Clare, 1996; Delauney, Compere & Lehaitre, 2010; Sánchez-Lozano et al., 2019). Based on the size, biofouling can be classified into microfouling, including bacteria and microalgae, and macrofouling, comprising macroalgae and sessile invertebrates (Clare, 1996; Briand, 2009; Delauney, Compere & Lehaitre, 2010), these latter ascribed to over 4,000 species (Yebra, Kiil & Dam-Johansen, 2004). Fouling communities are regulated by complex interactions between abiotic (temperature, conductivity, salinity, pH, dissolved oxygen, organic matter, hydrodynamics, light penetration and depth) and biotic (life cycle, reproduction time and strategy, larval lifespan, settlement, post-settlement competition and cooperation) factors (Almeida, Diamantino & de Sousa, 2007; Delauney, Compere & Lehaitre, 2010; Nandakumar & Yano, 2003). Indeed, significant seasonal variations were recorded in fouling colonization patterns, mostly depending on factors such as temperature, seawater dynamics and depth (Almeida, Diamantino & de Sousa, 2007; Whelan & Regan, 2006; Lezzi et al., 2018; Giangrande et al., 2020a).

In the past decades, there has been increasing interest in fouling because of its capability to cover all types of artificial substrates, including keels of boats, floating cages of mariculture and marine oil platforms (Castritsi-Catharios, Bourdaniotis & Persoone, 2007; Soliman, Mohamed & NaserGomaa, 2014). From the anthropocentric point of view, fouling produces numerous negative impacts as it increases the weight and drag of the submerged boat surfaces and, therefore, reduces the navigation efficiency. Moreover, fouling promotes corrosion of manufactured structures and also negatively affects mariculture, causing great economic losses of over 6,5 billion dollars per year (Eguía & Trueba, 2007; Lejars, Margaillan & Bressy, 2012; Limna Mol, Raveendran & Parameswaran, 2009; Ribeiro et al., 2013; Sánchez-Lozano et al., 2019; Tsoukatou et al., 2007; Vigneron, Head & Tsesmetzis, 2018; Giangrande et al., 2020a). Other major impacts of fouling are related to the introduction of alien species, which could be invasive and cause biodiversity loss or alter ecosystem functioning and services (Branch & Steffani, 2004; Lejars, Margaillan & Bressy, 2012; Ribeiro et al., 2013; Katsanevakis et al., 2014).

Such possible negative impacts arose many concerns and led to the development of various antifouling paints. These paints, based on toxic active ingredients, hampered the growth and cover of bacteria and sessile benthos but have had strong impacts on natural populations Hattori & Shizuri, 1996; Thomas & Brooks, 2010). The first antifouling paints were developed in the mid-nineteenth century and contained arsenic and mercury oxide as biocides. A century later, however, these latter were replaced with paints containing tributyltin (TBT). Although very efficient because of high toxicity (Almeida, Diamantino & De Sousa, 2007; Fernandez et al., 2005; Ribeiro et al., 2013), TBT compounds are well-known pollutants in the aquatic environment due to their toxic, persistent, bioaccumulative and endocrine disruptive characteristics (Antizar-Ladislao, 2008 and references therein). Indeed, International Maritime Organization adopted in 2008 an “International Convention on the Control of Harmful Anti-fouling Systems on Ships” which banned the use of organotin compounds in boat paints.

Alternative antifouling paints, based on non-pollutant compounds, can be obtained using effective natural products isolated from marine organisms, including macroalgae, sponges, gorgonians, bryozoans and ascidians (Liu, Wu & Qian, 2020), or synthetic compounds inspired by such natural products (Hellio et al., 2005; Limna Mol, Raveendran & Parameswaran, 2009; Soliman, Mohamed & NaserGomaa, 2014). In particular, many studies have been conducted on the antifouling activity of secondary metabolites produced by marine sessile organisms, that often use chemical defenses to keep their surface free from fouling (Almeida, Diamantino & de Sousa, 2007; Limna Mol, Raveendran & Parameswaran, 2009; Sánchez-Lozano et al., 2019). Marine sponges in particular, currently considered the top producers of active metabolites (Lee, Cho & Tran, 2021), have already been considered as possible reservoir of sustainable antifouling compounds (Müller et al., 2013). Nevertheless, little attention has been devoted until now to the class Calcarea.

With the aim of contributing to the development of bio-inspired antifouling paints harmless for the environment, the present study tested the antifouling effect of the ethanolic extract of the calcareous sponge *Paraleucilla magna* Klautau, Monteiro & Borojevic, 2004. The latter, reported for the first time as alien species for Mediterranean Sea by Longo, Mastrototaro & Corriero (2007), is rapidly expanding, with recent reports from both the central and eastern region of the basin (see Evcen & Çinar, 2020 and references therein). In the Mediterranean, *P. magna* mainly thrives in ports, marinas or inlets, on natural or artificial hard substrates, in photophilous or sciaphilous environments and in pristine or polluted waters (Longo, Mastrototaro & Corriero, 2007; Evcen & Çinar, 2020). The renowned ability of this sponge to infest native algae and filter-feeding invertebrates, described along Italian and Spanish coasts (Longo, Mastrototaro & Corriero, 2007; Guzzetti et al., 2019), makes it particularly interesting to test its potential as a producer of substances with antifouling properties.

The study was carried out on the population of *P. magna* from the seas of Taranto (southern Italy, northern Ionian Sea), where the species assumes invasive behavior thanks to rapid spreading capacities and considerable biomass production (Longo, Mastrototaro & Corriero, 2007). In particular, the settlement success of juvenile mussels (*Mytilus galloprovincialis*) was tested in the laboratory in the presence of different concentrations of sponge extract. The toxicity of this latter was tested on *Artemia salina*, one of the most used model species for ecotoxicological studies (Pecoraro et al., 2020), and in particular on its larvae (nauplii), very similar to the larvae of common encrusting organisms like the cyrripeds. Finally, the effects of the sponge extract were assessed on the marine microalgae *Nannochloropsis* sp. and *Tetraselmis suecica*, commonly cultivated in laboratory bioreactors and representative of those vegetal unicellular organisms characterizing the early stages of the biofouling colonization process (Müller et al., 2013).

## Materials and Methods

### Sponge collection

**Figure 1:**
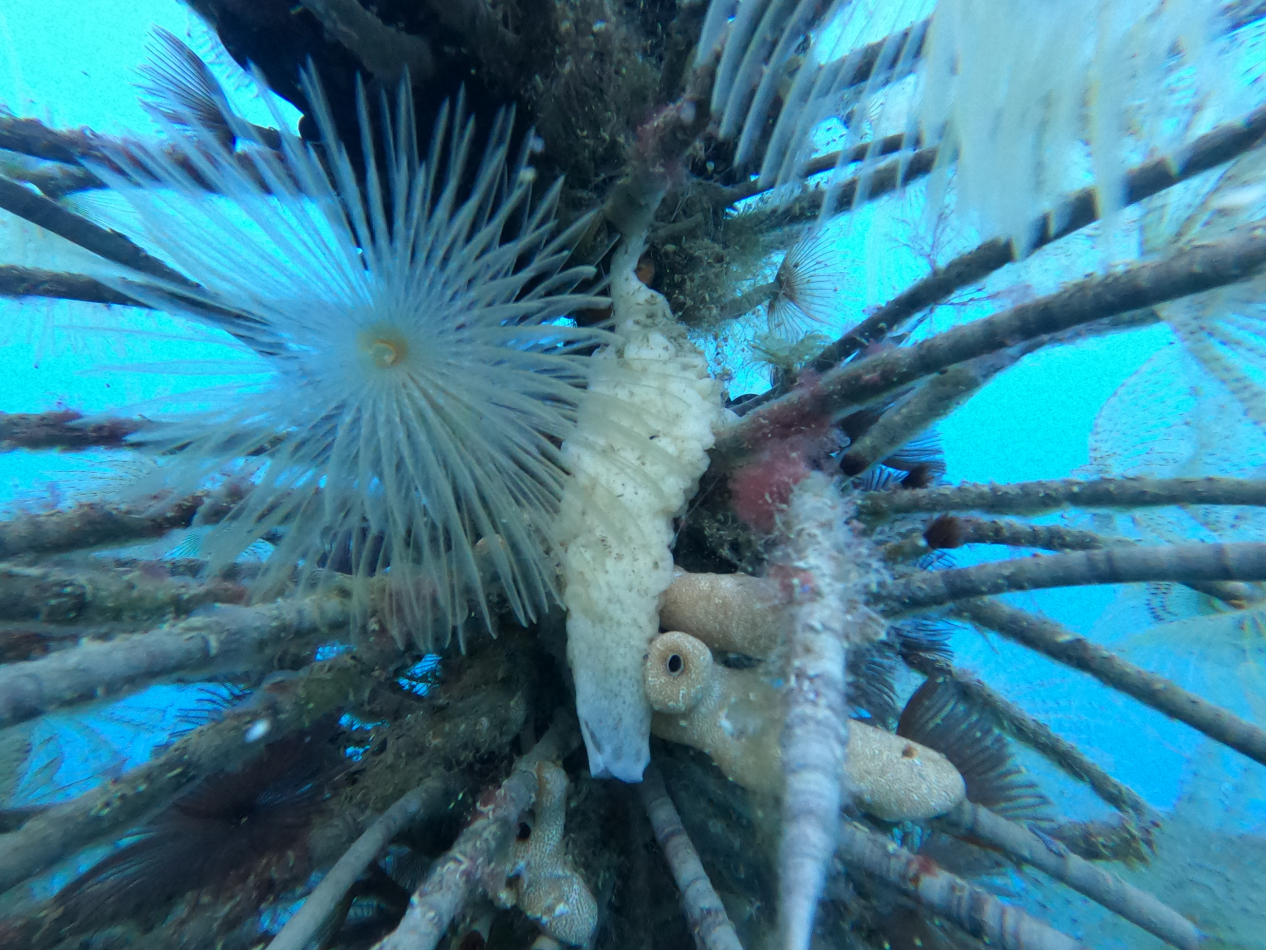
Sampling of *Paraleucilla magna*. Artificial substratum covered by polychaete tubes and ascidians, with a white specimen of *P. magna* in the foreground (photo credit: Roberta Trani).

Sponge specimens (1,500 g total wet weight) were collected in the Mar Grande of Taranto (40°26′N–17°14′E, Northern Ionian Sea) in January 2020. Sampling was performed by snorkeling from mussel rows and artificial substrata (Fig. 1). After collection, samples were placed in cooled bags and transported to the laboratory, where they were cleaned from associated fauna, rinsed with filtered sea water to remove debris and then freezed. Some fragments were randomly collected from the sponge biomass to validate the taxonomic identity of the samples (Longo, Mastrototaro & Corriero, 2007). No specific permits were required for field sample collection.

### Preparation of the sponge extract

Frozen sponge specimens were initially freeze-dried (224.53 g dry weight (DW)) and then extracted, through maceration in absolute ethanol (ethanol:sponge biomass = 10:1 mL/g) for 24 h. The whole procedure was repeated three times. The alcoholic extracts obtained were combined and concentrated with rotary vacuum pump at low temperatures (<40 °C). To obtain a water-soluble fraction of the extract for antifouling and toxicity tests, *Paraleucilla magna* ethanolic extract (5.24 g DW) was solubilized in 100 mL of distilled water by ultrasonic sonication and filtered under vacuum first with a Whatman I filter paper and then with a 0.22 µm pore size filters, until the water-insoluble part of the extract was completely removed (Hellio et al., 2005, with minor modifications). The water-soluble fraction obtained was lyophilized (4.38 g DW) and stored at −20 °C until its use in antifouling and toxicity tests. Before starting the experiments, a stock solution (100 g/L) of this water-soluble extract was prepared by dissolving the dry extract in artificial sea water (ASW) (Aquaforest^®^ Sea Salt, 37 practical salinity units (psu)) and stirring the stock solution until the extract is completely dissolved. The stock solution was diluted with ASW to prepare the different extract concentrations tested.

### Antifouling assays with *Mytilus galloprovincialis*

Juveniles (less than one year from larval settlement) of *Mytilus galloprovincialis* Lamarck, 1819 were collected during low tide from rocky coastal bottoms of the Mar Piccolo of Taranto (40°29′N–17°19′E). No specific permits were required for field sample collection. Specimens were transported to the laboratory and maintained in aerated aquaria with ASW at 18 ± 2  °C and 37 psu for one night (Kitajima et al., 1995). After acclimation, mussels were put in a plastic plate filled with ASW, separated from each other by carefully cutting the byssus filaments and finally measured. Specimens with a total shell length between 6 and 9 mm able to exhibit a substrate exploring behavior, i.e., actively exposing their foot and crawling, were then selected for the experiment (Kitajima et al., 1995; Da Gama et al., 2003).

**Figure 2:**
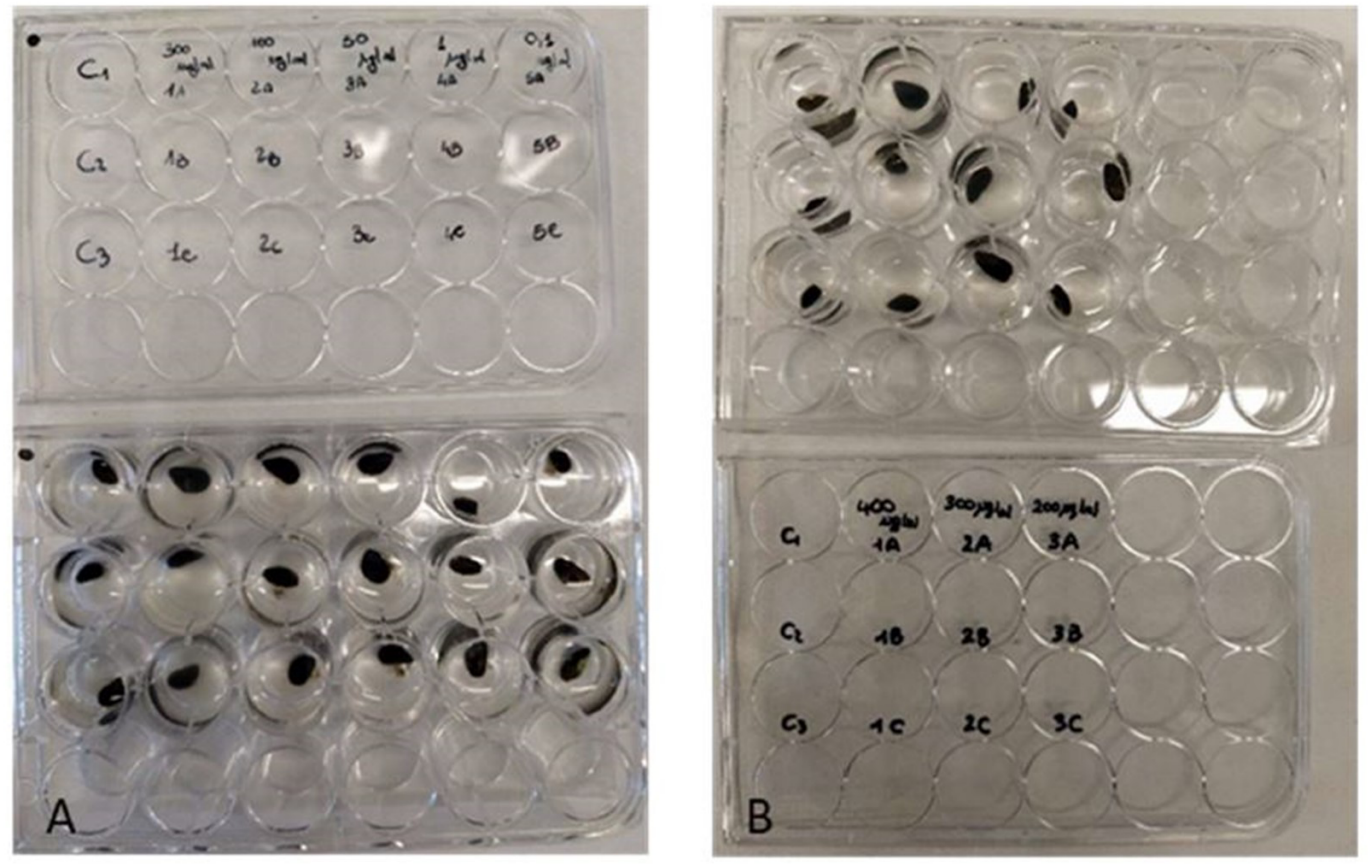
Antifouling tests on *Mytilus galloprovincialis*. Multi-well plates with one specimen of *M. galloprovincialis* for each well. The mussels were kept at different concentrations of *P. magna* extract or in artificial sea water alone (control), during Test 1 (A) and Test 2 (B) (photo credit: Roberta Trani).

To determine the antifouling activity on juvenile mussels, the *P. magna* extract was initially tested (Test 1) at five increasing concentrations (0.1, 1, 50, 100 and 300 µg/mL). According to the results obtained in this first phase, a second test was carried out using concentrations of 200, 300 and 400 µg/mL (Test 2). All the experiments were carried out in multi-well plates (Fig. 2), placing one individual of *M. galloprovincialis* in each well, containing 2 ml of ASW with *P. magna* extract at a different concentration, and considering three individuals for each concentration. Control experiments were performed using only ASW. The experiment lasted for 72 h. Plates were incubated in the dark and at a temperature of 20 °C, following previous studies showing these conditions as optimal for the active production of byssal filaments (Da Gama et al., 2003). At 24-hour intervals, the number of individuals who adhered to the substrate was counted.

At the end of each experiment, the viability of mussels that showed no adhesion was assessed. These individuals were placed in wells that contained only 2 ml of ASW and were held under the same conditions as in antifouling assays. Live individuals were counted after 24 h.

### Toxicity assessment of the sponge extract by bioassay on brine shrimps

**Figure 3:**
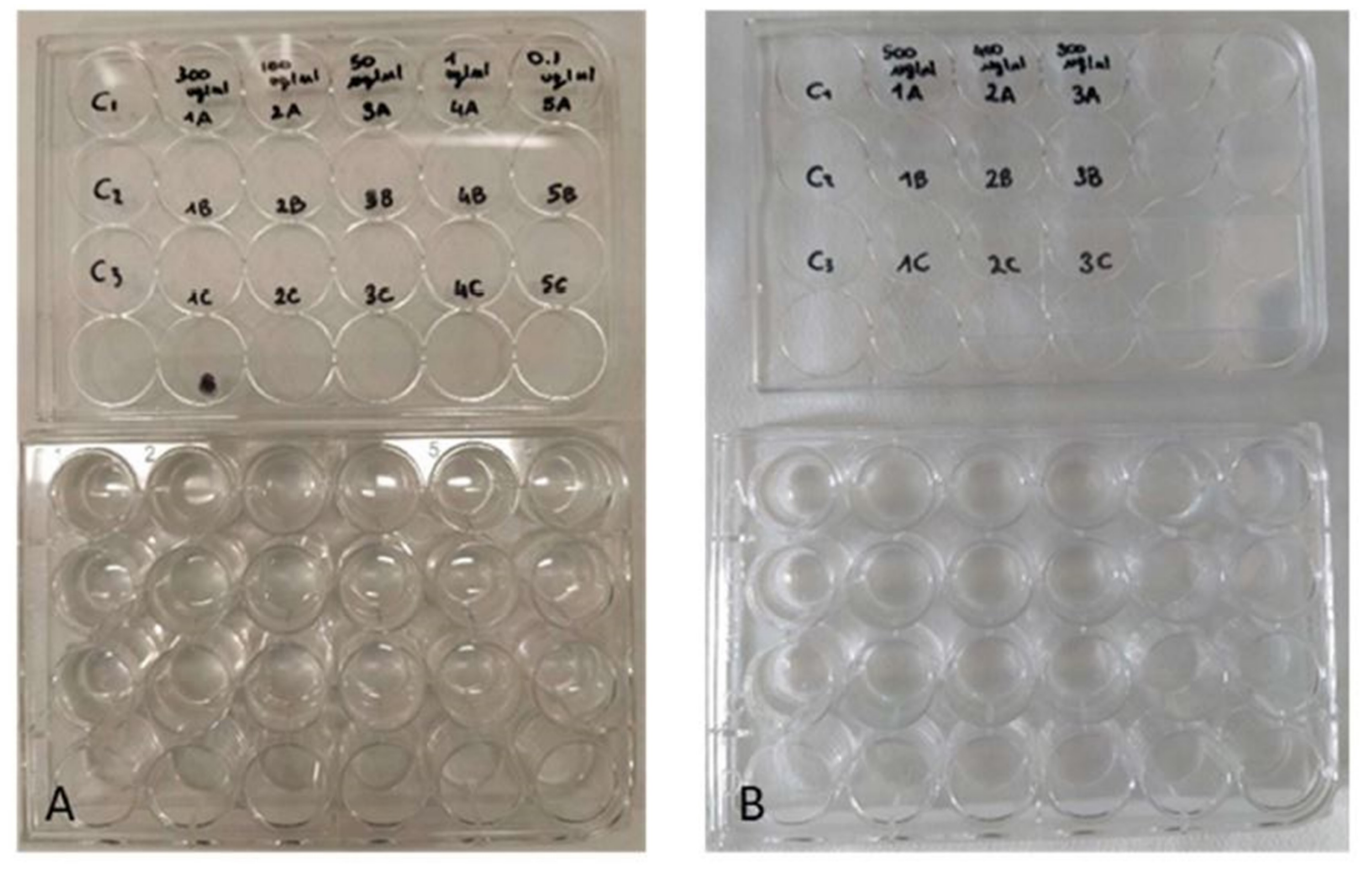
Toxicity tests on *Artemia salina* nauplii. Multi-well plates containing nauplii of *A. salina*. The nauplii were kept at different concentrations of *P. magna* extract or in artificial sea water alone (control), during Test 1 (A) and Test 2 (B) (photo credit: Roberta Trani).

The toxicity of the ethanolic sponge extract was tested on the larvae (nauplii) of the brine shrimp *Artemia salina* (Linnaeus, 1758). With this aim, dehydrated cysts of *A. salina* (SHG, Premium Artemia cysts) were incubated in a hatching chamber filled with ASW (25 °C, 33 psu). After 48 h, the nauplii were collected with a Pasteur pipette and used for toxicity assays (Castritsi-Catharios, Bourdaniotis & Persoone, 2007; Lagarto Parra et al., 2001). A first test was carried out with the concentrations 0.1, 1, 50, 100 and 300 µg/mL, followed by a second test with the concentrations 300, 400 and 500 µg/mL. Control experiments were performed using only ASW. The experiments were performed in triplicates in multi-well plates (Fig. 3). Each well contained four nauplii of *A. salina* in a final volume of two mL. Plates were incubated for 48 h at 25 °C. Live nauplii were counted every 24 h under a dissecting microscope. The nauplii of *A. salina* were considered dead if they showed no movement during a 30 s observation period (Castritsi-Catharios, Bourdaniotis & Persoone, 2007; Lagarto Parra et al., 2001; Sánchez-Lozano et al., 2019).

### Toxicity assessment of the sponge extract by bioassay on microalgae

**Figure 4:**
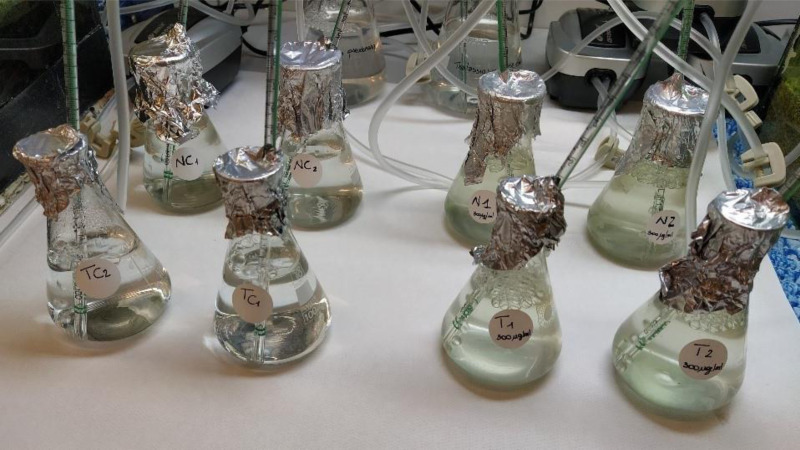
Toxicity tests on *Nannochloropsis* sp. and *Tetraselmis suecica*. Flasks containing cultures of the algal species *Nannochloropsis* sp. (back row) and *T. suecica* (front row) (N1-2 and T1-2 = treatments; NC1-2 and TC1-2 = controls) (photo credit: Roberta Trani).

Microalgal strains were obtained directly from photobioreactors of the Biology Department of Bari University. In particular, the experimentation was carried out on the species *Nannochloropsis* sp. and *Tetraselmis suecica* (Kylin) Butcher, 1959. For each species, four sterile glass flasks containing 100 mL of algal culture (5  × 10^5^ cells/mL) were prepared. The *P. magna* extract at the concentration of 300 µg/mL was added to two of the flasks (treatments), while the other two, containing no sponge extract, were used as a control for algal growth (control, C) (Fig. 4). The culture medium was enriched with 1.5 mL/L of ARD2 and ENW fertilizers for *Nannochloropsis* sp. and *T. suecica*, respectively.

Flasks were incubated for five days at room temperature (18 ± 2 °C). They were provided with artificial illumination (light:dark 16:8 h, white lamp) and continuous aeration, and were covered with aluminum foil to avoid evaporation and external contamination (Dupraz et al., 2018).

Algal growth was measured by cell counts using the Bürker chamber (Faimali et al., 2003; Magnusson, Heimann & Negri, 2008). Counting was performed at 24, 96 and 120 h.

### Statistical analysis

A PERMANOVA (PERmutational Multivariate ANalysis Of VAriance) non-parametric statistic test (Anderson, 2001) was used to evaluate the significance of the results obtained using different concentrations of the *P. magna* extract. The tests were run on Bray–Curtis similarity matrices with 9999 permutations (Anderson, 2001). The concentrations of the *P. magna* extract (C, 9 levels) and exposure time (t, 4 levels) were the factors used to detect differences in the toxicity tests on *A. salina* nauplii and on the two microalgae *Nannochloropsis* sp. and *T. suecica,* in C × t interactions. Each interaction was individually analysed using Univariate PERMANOVA tests with the same experimental design. A pairwise test was applied to discover statistically significant differences in each pair of factor levels based on the significance value of PERMANOVA/Monte Carlo tests. Differences were considered to be significant for *p* < 0.05. All analyses were conducted using Primer v6+ PERMANOVA software (Anderson, Gorley & Clarke, 2008).

## Results

### Antifouling bioassays

**Figure 5:**
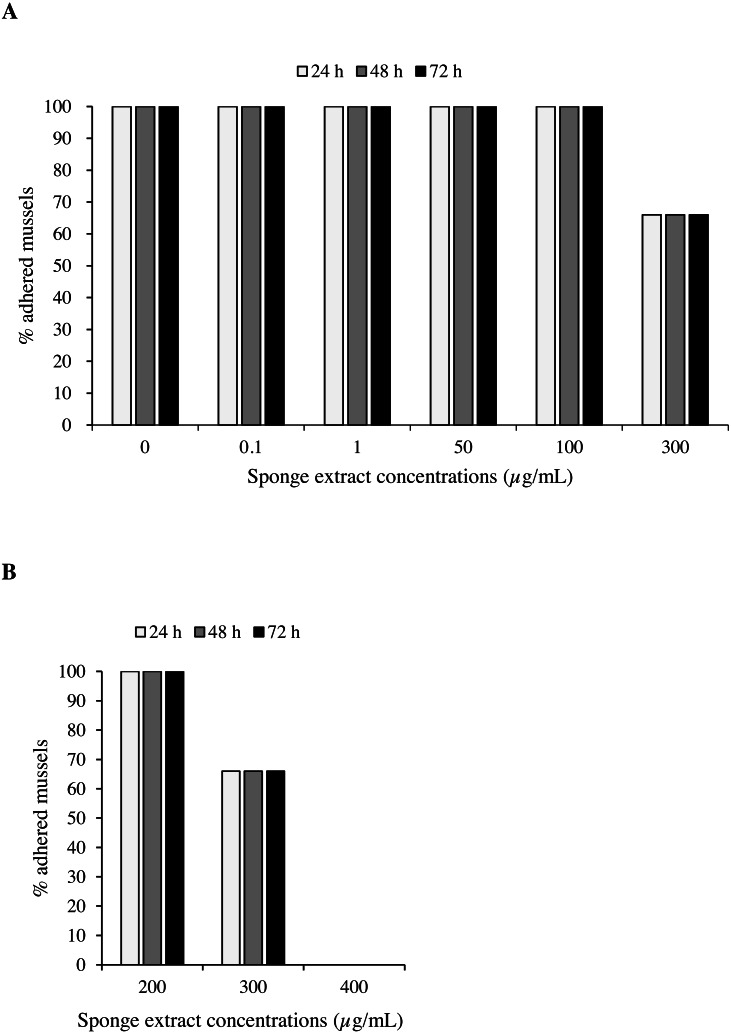
Antifouling tests on *Mytilus galloprovincialis*. Percentage of adhered specimens of *M. galloprovincialis* after treatment with different concentrations of *P. magna* ethanolic extract during Test 1 (A) and Test 2 (B), at each observation time (24, 48 and 72 h).

Mussels exposed to the sponge extract at a concentration below 300 µg/mL, including controls, showed undisturbed settlement. Extract at a concentration of 300 µg/mL led to a 33.3% reduction in mussel settling (2 detached mussels over 6) after 24 h, with no further reductions observed thereafter (Fig. 5). At the concentration of 400 µg/mL (Test 2), settling inhibition was observed in 100% of the individuals after 24 h from the beginning of the experiment and no production of byssus was recorded. To evaluate the viability at the end of these latter experiments, unattached individuals were transferred into plates with ASW only and were held under the same experimental conditions for 24 h. Subsequent observation under the dissecting microscope revealed adhesion of 100% of the mussels.

### Toxicity bioassay with brine shrimp nauplii

**Figure 6:**
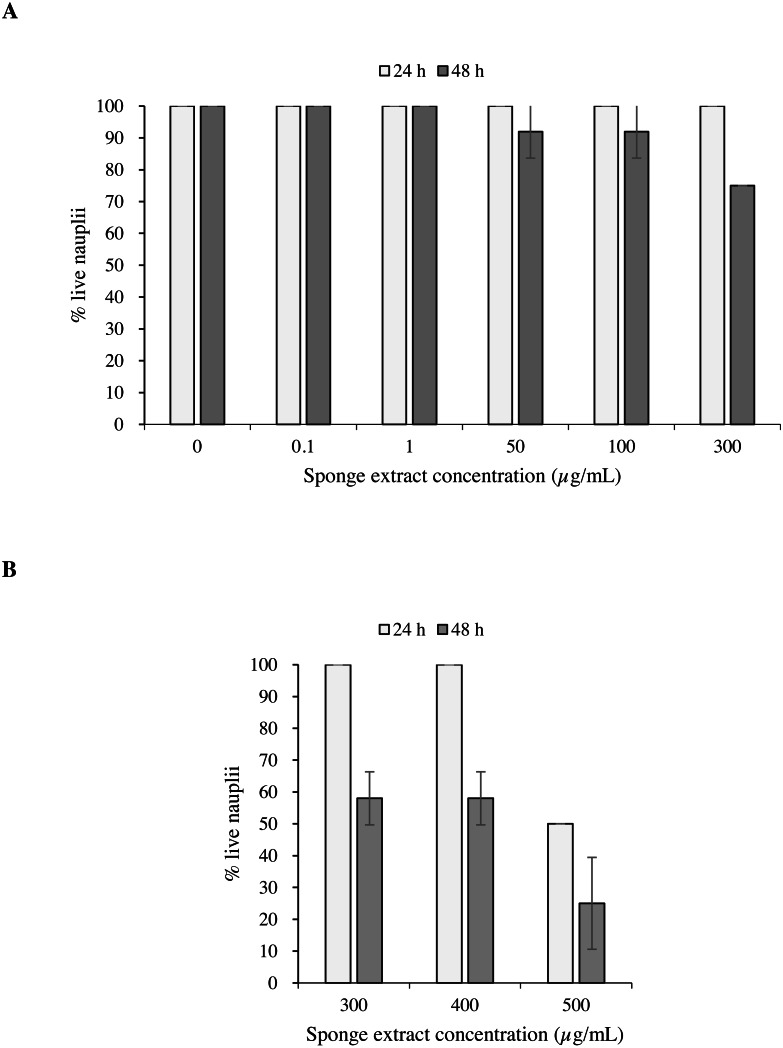
Toxicity tests on *Artemia salina* nauplii. Percentage of live *A. salina* nauplii exposed to different concentrations of *P. magna* ethanolic extract at each observation time (24 and 48 h) during Test 1 (A) and Test 2 (B). Error bars indicate standard errors.

Test 1 carried out on the nauplii of *Artemia salina* with concentrations 0.1, 50, 100 and 300 µg/mL of the sponge extract showed no mortality after 24 h (Fig. 6A). After 48 h, however, the observed survival was 91.7 ± 8.3% (mean ±  standard error) in presence of extract concentrations of 50 and 100 µg/mL, while at the concentration of 300 µg/mL survival decreased to 75.0 ± 0.0%. Test 2 showed 50.0 ±  0.0% of survival of the nauplii at a concentration of 500 µg/mL after 24 h from the beginning of the test. This result can also be expressed as Lethal Concentration for 50% of the nauplii (LC50) (Fig. 6B).

The results of the Univariate PERMANOVA non-parametric statistical test showed that the percentage of live *A. salina* nauplii was affected by the concentration of *Paraleucilla magna* extract (C), the exposure time (t) and their interaction (C × t) (univariate PERMANOVA, pseudo-F = 2.9188, *df* = 14, *p* = 0.0001). The pairwise comparison as a function of the extract concentration highlighted a significant decrease in the percentage of live nauplii starting from 300 µg/mL after 48 h of exposure (*p* < 0.01) and at 500 µg/mL after 24 h (*p* < 0.05).

### Toxicity bioassays: inhibition of microalgal growth

**Figure 7:**
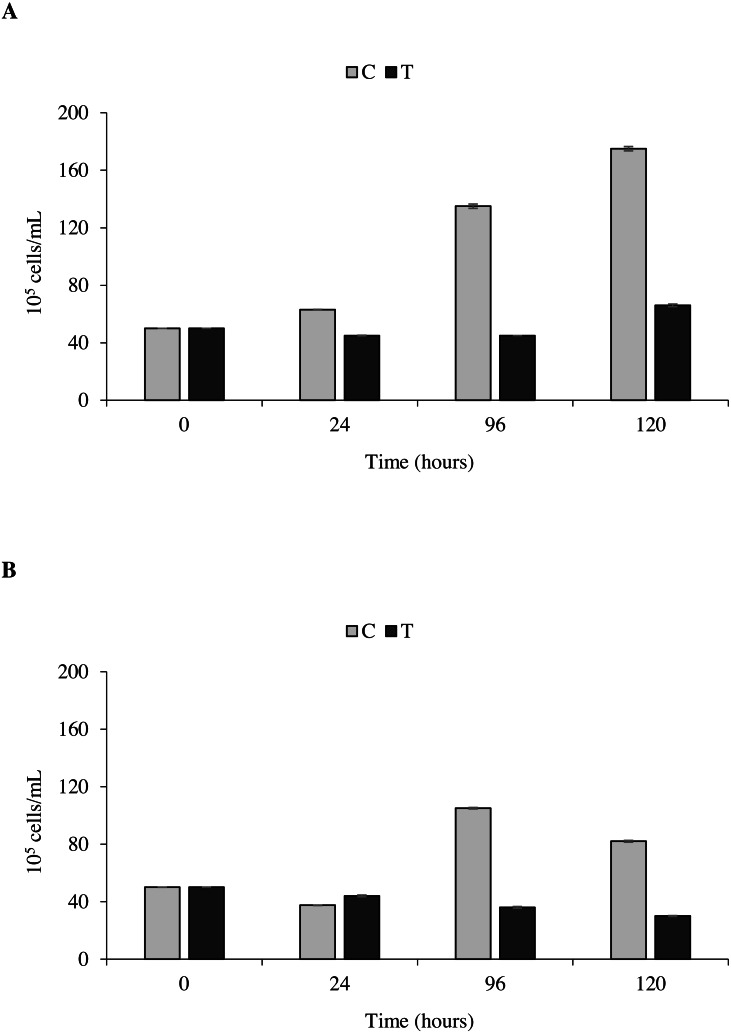
Toxicity tests on *Nannochloropsis.* sp. and *Tetraselmis suecica*. Cell density of *Nannochloropsis* sp. (A) and *T. suecica* (B) exposed to 300 µg/mL of *P. magna* ethanolic extract, at each observation time (0, 24, 96 and 120 h) (T), compared to the control (C). Error bars indicate standard errors.

The toxicity test of *P. magna* extract (300 µg/mL) on microalgae of the species *Nannochloropsis* sp. and *Tetraselmis suecica* was aimed at measuring a possible inhibition of their growth. Figure 7A shows that in the control flasks containing *Nannochloropsis* sp. an increase in cell density as a function of time occurred, reaching the maximum value of 1.9 × 10^6^ cells/mL after 120 h from the beginning of the experiment. In the treatments, a 10.0 ± 1.6% decrease of cell density was observed at 24 and 96 h, followed by a slight increase at 120 h, though with values always lower than the control. Similarly, in the control flasks containing *T. suecica* an increase in cell density was recorded, with a maximum value of 1.1 × 10^6^ cells/mL reached 96 h after the beginning of the experiment, followed by a 22.0 ± 2.0% decrease at 120 h. A significant inhibition (*p* < 0.05) of algal growth was observed in the treatment flasks containing *T. suecica*, with a 40.0 ± 6.0% decrease in cell density between the initial and final concentrations (Fig. 7B).

Statistical analysis confirms that the density growth over time of both tested algal species is significantly influenced by the presence of the *P. magna* extract (univariate PERMANOVA, *Nannochloropsis* sp.: *df* = 3, pseudo-F = 29.833, *p* = 0.0001; *T. suecica*: *df* = 3, pseudo-F = 21.849, *p* = 0.0011).

## Discussion

Several studies have investigated the inhibitory effect exerted on marine fouling organisms by sponges, sessile invertebrates that must compete for space during their whole life cycle. A research by Tsoukatou et al. (2002) carried out in Mediterranean Sea on sponges of the family Irciniidae showed significant inhibition of mussel settlement (68%) and algal growth (>60%) caused by an extract of *Ircinia oros* (Schmidt, 1864) at the concentration of 30 µg/mL. Methanolic extracts of *Haliclona (Soestella) caerulea* (Hechtel, 1965) and *Ircinia* sp. at concentrations of 200 µg/mL prevented mussel settlement (35–45%), while extracts of the algae *Sargassum horridum* Setchell & N.L. Gardner, 1924 and *Laurencia gardneri* Hollenberg, 1943 inhibited 95–100% of mussel settlement and caused 50% mortality in *Artemia salina* nauplii at a concentration higher than 1,000 µg/mL (Sánchez-Lozano et al., 2019). Further studies, focusing on barnacles, demonstrated the antifouling activity of different Mediterranean sponge extracts (*I. oros*, *Sarcotragus spinosulus* Schmidt, 1862, *Dysidea* sp., *Scalarispongia scalaris* (Schmidt, 1862), *Hippospongia communis* (Lamarck, 1814), with about 60% of barnacle settlement inhibition at a concentration of 100 µg/mL (Hellio et al., 2005).

The results of the present work contribute to increasing the knowledge on the natural alternatives to toxic antifouling products. Indeed, the ethanolic extract of the calcareous sponge *Paraleucilla magna* (alien in Mediterranean Sea) inhibited settlement of juvenile *Mytilus galloprovincialis* and demonstrated toxic effects on the nauplii of *A. salina* and on the microalgae *Nannochloropsis* sp. and *Tetraselmis suecica*. At a concentration of 300 µg/mL, the sponge extract prevented 30% of *M. galloprovincialis* individuals from adhering to the substrate, while at a concentration of 400 µg/mL it led to the complete inhibition of mussel settlement, demonstrating remarkable antifouling properties. However, the same mussels that were prevented from settling, once transferred to sea water without sponge extract, returned to adhere to the substrate, revealing a reversible mechanism of action of the antifouling metabolites present in the extract of *P. magna*, similar to that described for other natural compounds with antifouling properties (Göransson et al., 2004).

To fully comprehend the potential applications of the sponge extract in marine antifouling paints, its toxicity was tested on three additional model organisms. In particular, tests on *A. salina* nauplii provided highly significant results and demonstrated moderate or high toxicity, depending on the concentration applied. Extracts at concentrations of 300 and 400 µg/mL caused mortality of more than 40% of the nauplii after 48 h of exposure, while a concentration of 500 µg/mL killed 50% of the nauplii after only 24 h from the beginning of the experiment. Although *A. salina* is not a component of marine fouling, it is one of the most used model species for toxicity studies (Pecoraro et al., 2020). Moreover, the toxicity revealed by the present study on the nauplii of this species may suggest that the alcoholic extract of *P. magna* could also have effects on larvae of typically encrusting species, such as sea squirts and bryozoans.

At the concentration of 300 µg/mL the extract of *P. magna* had a considerable inhibitory effect on the growth of *Nannochloropsis* sp. and *T. suecica*. Indeed, during the entire experiment, the number of cells of these two microalgae remained constantly low when compared to the controls, in which densities were more than doubled after 96 h. However, it should be underlined that algal cells remained viable, although unable to grow.

Following the results of the present study, it is reasonable to assume that *Paraleucilla magna* ethanolic extract contains metabolites with antifouling activity exerting a deterrent effect on both animal and vegetal organisms. Such metabolites proved to be effective in preventing mussel settling and inhibiting microalgal growth, this latter usually responsible of forming a film that facilitates further substrate colonization (Müller et al., 2013). Furthermore, these compounds have a dose-dependent toxic effect on the nauplii of the crustacean *A. salina*, suggesting that they can have a similar effect on the nauplii of other crustaceans such as the cyrripeds, renown protagonists of fouling communities worldwide (Göransson et al., 2004).

The extract of *P. magna* could therefore be advantageously included in the composition of antifouling paints. However, since laboratory conditions make it difficult to exactly reproduce the multiple physical, chemical and biological interactions that characterize natural ecosystems (Bressy et al., 2010), experiments will be performed in the future to test the efficacy of this extract in the field. In addition, research will be conducted to isolate and chemically characterize the active compounds within the extract of *P. magna*.

*P. magna* is considered an alien and invasive species in the Mediterranean Sea (Longo, Mastrototaro & Corriero, 2007; Evcen & Çinar, 2020), where it locally harms the activity of mussel farmers who make a considerable effort to control its growth (Longo, Mastrototaro & Corriero, 2007). However, it appears that some introduced species may have positive effects on the new environment they have colonized (Giangrande et al., 2020b), sometimes becoming providers of ecosystem services. In this scenario, the discovery of antifouling metabolites in *P. magna* can bring economic benefits to the local fishermen who will exploit the resource. Furthermore, the collection of the sponge for the extraction of antifouling compounds would automatically contribute to the control of its abundance in the coastal ecosystems of the Mediterranean Sea.

## Supplemental Information

10.7717/peerj.12279/supp-1 Supplemental Information 1 Raw data

## Additional Information and Declarations

### Field Study Permissions

The following information was supplied relating to field study approvals (i.e., approving body and any reference numbers): No specific permits were required for the described field studies.

### Data Availability

The following information was supplied regarding data availability: The raw data regarding the live *Artemia salina* nauplii counts and micro algae cells counts are reported in the File S1.
